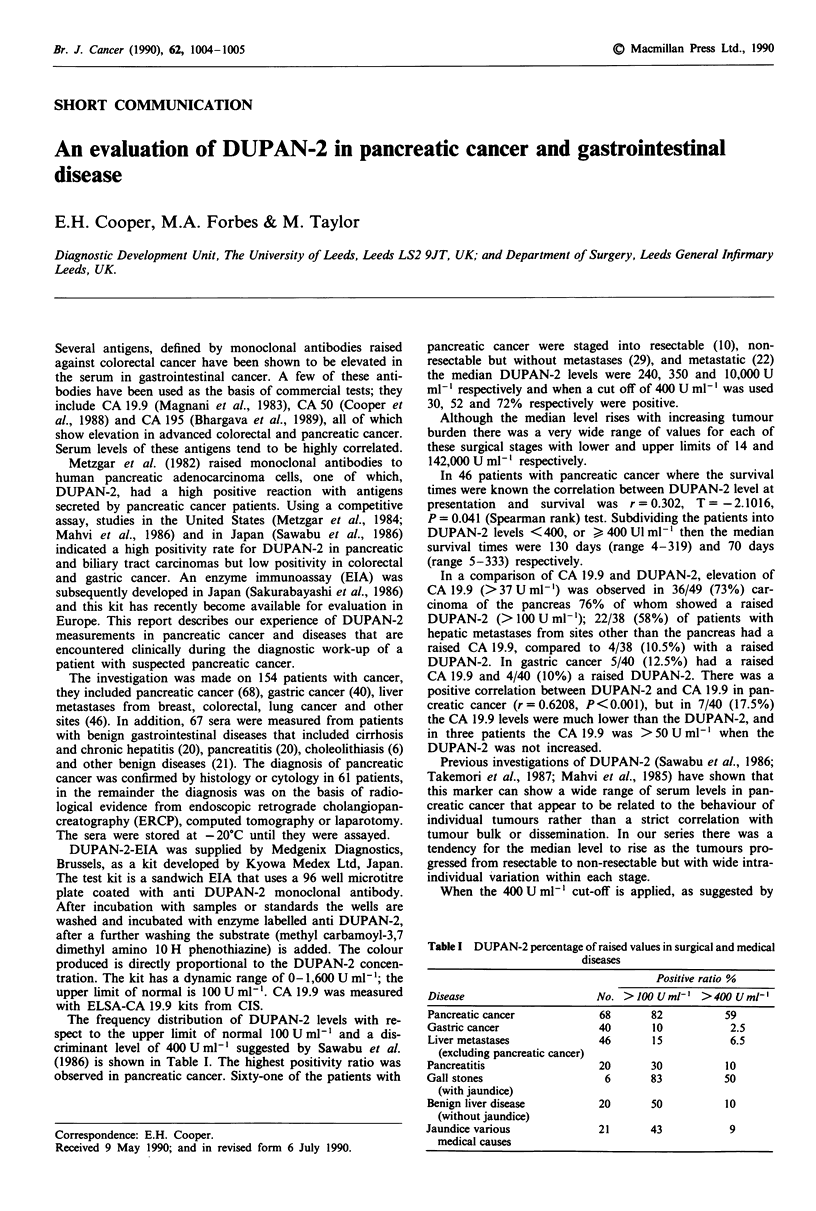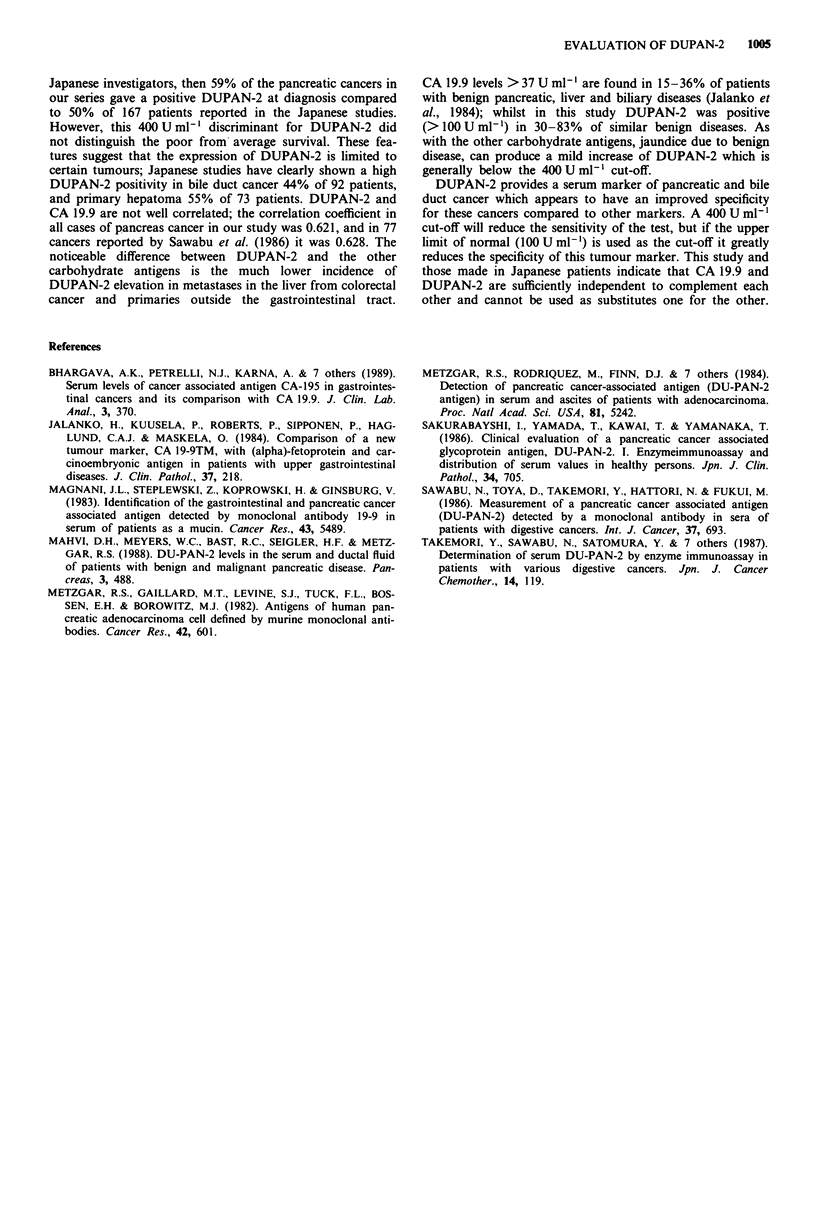# An evaluation of DUPAN-2 in pancreatic cancer and gastrointestinal disease.

**DOI:** 10.1038/bjc.1990.426

**Published:** 1990-12

**Authors:** E. H. Cooper, M. A. Forbes, M. Taylor

**Affiliations:** Diagnostic Development Unit, University of Leeds, UK.


Br. J. Cancer (1990), 62, 1004–1005 © Macmillan Press Ltd., 1990

SHORT COMMUNICATION

### An evaluation of DUPAN-2 in pancreatic cancer and gastrointestinal disease

E.H. Cooper, M.A. Forbes & M. Taylor

*Diagnostic Development Unit, The University of Leeds, Leeds LS2 9JT, UK; and Department of Surgery, Leeds General Infirmary Leeds, UK.*

Several antigens, defined by monoclonal antibodies raised against colorectal cancer have been shown to be elevated in the serum in gastrointestinal cancer. A few of these antibodies have been used as the basis of commercial tests; they include CA 19.9 (Magnani *et al.*, 1983), CA 50 (Cooper *et al.*, 1988) and CA 195 (Bhargava *et al.*, 1989), all of which show elevation in advanced colorectal and pancreatic cancer. Serum levels of these antigens tend to be highly correlated.

Metzgar *et al.* (1982) raised monoclonal antibodies to human pancreatic adenocarcinoma cells, one of which, DUPAN-2, had a high positive reaction with antigens secreted by pancreatic cancer patients. Using a competitive assay, studies in the United States (Metzgar *et al.*, 1984; Mahvi *et al.*, 1986) and in Japan (Sawabu *et al.*, 1986) indicated a high positivity rate for DUPAN-2 in pancreatic and biliary tract carcinomas but low positivity in colorectal and gastric cancer. An enzyme immunoassay (EIA) was subsequently developed in Japan (Sakurabayashi *et al.*, 1986) and this kit has recently become available for evaluation in Europe. This report describes our experience of DUPAN-2 measurements in pancreatic cancer and diseases that are encountered clinically during the diagnostic work-up of a patient with suspected pancreatic cancer.

The investigation was made on 154 patients with cancer, they included pancreatic cancer (68), gastric cancer (40), liver metastases from breast, colorectal, lung cancer and other sites (46). In addition, 67 sera were measured from patients with benign gastrointestinal diseases that included cirrhosis and chronic hepatitis (20), pancreatitis (20), choleolithiasis (6) and other benign diseases (21). The diagnosis of pancreatic cancer was confirmed by histology or cytology in 61 patients, in the remainder the diagnosis was on the basis of radiological evidence from endoscopic retrograde cholangiopancreatography (ERCP), computed tomography or laparotomy. The sera were stored at −20°C until they were assayed.

DUPAN-2-EIA was supplied by Medgenix Diagnostics, Brussels, as a kit developed by Kyowa Medex Ltd, Japan. The test kit is a sandwich EIA that uses a 96 well microtitre plate coated with anti DUPAN-2 monoclonal antibody. After incubation with samples or standards the wells are washed and incubated with enzyme labelled anti DUPAN-2, after a further washing the substrate (methyl carbamoyl-3,7 dimethyl amino 10 H phenothiazine) is added. The colour produced is directly proportional to the DUPAN-2 concentration. The kit has a dynamic range of 0–1,600 U $ml^{-1}$; the upper limit of normal is 100 U $ml^{-1}$. CA 19.9 was measured with ELSA-CA 19.9 kits from CIS.

The frequency distribution of DUPAN-2 levels with respect to the upper limit of normal 100 U $ml^{-1}$ and a discriminant level of 400 U $ml^{-1}$ suggested by Sawabu *et al.* (1986) is shown in Table I. The highest positivity ratio was observed in pancreatic cancer. Sixty-one of the patients with pancreatic cancer were staged into resectable (10), non-resectable but without metastases (29), and metastatic (22) the median DUPAN-2 levels were 240, 350 and 10,000 U $ml^{-1}$ respectively and when a cut off of 400 U $ml^{-1}$ was used 30, 52 and 72% respectively were positive.

Although the median level rises with increasing tumour burden there was a very wide range of values for each of these surgical stages with lower and upper limits of 14 and 142,000 U $ml^{-1}$ respectively.

In 46 patients with pancreatic cancer where the survival times were known the correlation between DUPAN-2 level at presentation and survival was $r = 0.302$, $T = -2.1016$, $P = 0.041$ (Spearman rank) test. Subdividing the patients into DUPAN-2 levels <400, or ⩾400 Ul $ml^{-1}$ then the median survival times were 130 days (range 4–319) and 70 days (range 5–333) respectively.

In a comparison of CA 19.9 and DUPAN-2, elevation of CA 19.9 (>37 U $ml^{-1}$) was observed in 36/49 (73%) carcinoma of the pancreas 76% of whom showed a raised DUPAN-2 (>100 U $ml^{-1}$); 22/38 (58%) of patients with hepatic metastases from sites other than the pancreas had a raised CA 19.9, compared to 4/38 (10.5%) with a raised DUPAN-2. In gastric cancer 5/40 (12.5%) had a raised CA 19.9 and 4/40 (10%) a raised DUPAN-2. There was a positive correlation between DUPAN-2 and CA 19.9 in pancreatic cancer ($r = 0.6208$, $P < 0.001$), but in 7/40 (17.5%) the CA 19.9 levels were much lower than the DUPAN-2, and in three patients the CA 19.9 was >50 U $ml^{-1}$ when the DUPAN-2 was not increased.

Previous investigations of DUPAN-2 (Sawabu *et al.*, 1986; Takemori *et al.*, 1987; Mahvi *et al.*, 1985) have shown that this marker can show a wide range of serum levels in pancreatic cancer that appear to be related to the behaviour of individual tumours rather than a strict correlation with tumour bulk or dissemination. In our series there was a tendency for the median level to rise as the tumours progressed from resectable to non-resectable but with wide intra-individual variation within each stage.

When the 400 U $ml^{-1}$ cut-off is applied, as suggested by

**Table I** DUPAN-2 percentage of raised values in surgical and medical diseases

| | | Positive ratio % | |
|---|---|---|---|
| *Disease* | *No.* | *>100 U $ml^{-1}$* | *>400 U $ml^{-1}$* |
| Pancreatic cancer | 68 | 82 | 59 |
| Gastric cancer | 40 | 10 | 2.5 |
| Liver metastases (excluding pancreatic cancer) | 46 | 15 | 6.5 |
| Pancreatitis | 20 | 30 | 10 |
| Gall stones (with jaundice) | 6 | 83 | 50 |
| Benign liver disease (without jaundice) | 20 | 50 | 10 |
| Jaundice various medical causes | 21 | 43 | 9 |

Correspondence: E.H. Cooper.
Received 9 May 1990; and in revised form 6 July 1990.

Japanese investigators, then 59% of the pancreatic cancers in our series gave a positive DUPAN-2 at diagnosis compared to 50% of 167 patients reported in the Japanese studies. However, this 400 U $ml^{-1}$ discriminant for DUPAN-2 did not distinguish the poor from average survival. These features suggest that the expression of DUPAN-2 is limited to certain tumours; Japanese studies have clearly shown a high DUPAN-2 positivity in bile duct cancer 44% of 92 patients, and primary hepatoma 55% of 73 patients. DUPAN-2 and CA 19.9 are not well correlated; the correlation coefficient in all cases of pancreas cancer in our study was 0.621, and in 77 cancers reported by Sawabu *et al.* (1986) it was 0.628. The noticeable difference between DUPAN-2 and the other carbohydrate antigens is the much lower incidence of DUPAN-2 elevation in metastases in the liver from colorectal cancer and primaries outside the gastrointestinal tract. CA 19.9 levels $>37$ U $ml^{-1}$ are found in 15–36% of patients with benign pancreatic, liver and biliary diseases (Jalanko *et al.*, 1984); whilst in this study DUPAN-2 was positive ($>100$ U $ml^{-1}$) in 30–83% of similar benign diseases. As with the other carbohydrate antigens, jaundice due to benign disease, can produce a mild increase of DUPAN-2 which is generally below the 400 U $ml^{-1}$ cut-off.

DUPAN-2 provides a serum marker of pancreatic and bile duct cancer which appears to have an improved specificity for these cancers compared to other markers. A 400 U $ml^{-1}$ cut-off will reduce the sensitivity of the test, but if the upper limit of normal (100 U $ml^{-1}$) is used as the cut-off it greatly reduces the specificity of this tumour marker. This study and those made in Japanese patients indicate that CA 19.9 and DUPAN-2 are sufficiently independent to complement each other and cannot be used as substitutes one for the other.

### References

BHARGAVA, A.K., PETRELLI, N.J., KARNA, A. & 7 others (1989). Serum levels of cancer associated antigen CA-195 in gastrointestinal cancers and its comparison with CA 19.9. *J. Clin. Lab. Anal.*, **3**, 370.

JALANKO, H., KUUSELA, P., ROBERTS, P., SIPPONEN, P., HAGLUND, C.A.J. & MASKELA, O. (1984). Comparison of a new tumour marker, CA 19-9TM, with (alpha)-fetoprotein and carcinoembryonic antigen in patients with upper gastrointestinal diseases. *J. Clin. Pathol.*, **37**, 218.

MAGNANI, J.L., STEPLEWSKI, Z., KOPROWSKI, H. & GINSBURG, V. (1983). Identification of the gastrointestinal and pancreatic cancer associated antigen detected by monoclonal antibody 19-9 in serum of patients as a mucin. *Cancer Res.*, **43**, 5489.

MAHVI, D.H., MEYERS, W.C., BAST, R.C., SEIGLER, H.F. & METZGAR, R.S. (1988). DU-PAN-2 levels in the serum and ductal fluid of patients with benign and malignant pancreatic disease. *Pancreas*, **3**, 488.

METZGAR, R.S., GAILLARD, M.T., LEVINE, S.J., TUCK, F.L., BOSSEN, E.H. & BOROWITZ, M.J. (1982). Antigens of human pancreatic adenocarcinoma cell defined by murine monoclonal antibodies. *Cancer Res.*, **42**, 601.

METZGAR, R.S., RODRIQUEZ, M., FINN, D.J. & 7 others (1984). Detection of pancreatic cancer-associated antigen (DU-PAN-2 antigen) in serum and ascites of patients with adenocarcinoma. *Proc. Natl Acad. Sci. USA*, **81**, 5242.

SAKURABAYSHI, I., YAMADA, T., KAWAI, T. & YAMANAKA, T. (1986). Clinical evaluation of a pancreatic cancer associated glycoprotein antigen, DU-PAN-2. I. Enzymeimmunoassay and distribution of serum values in healthy persons. *Jpn. J. Clin. Pathol.*, **34**, 705.

SAWABU, N., TOYA, D., TAKEMORI, Y., HATTORI, N. & FUKUI, M. (1986). Measurement of a pancreatic cancer associated antigen (DU-PAN-2) detected by a monoclonal antibody in sera of patients with digestive cancers. *Int. J. Cancer*, **37**, 693.

TAKEMORI, Y., SAWABU, N., SATOMURA, Y. & 7 others (1987). Determination of serum DU-PAN-2 by enzyme immunoassay in patients with various digestive cancers. *Jpn. J. Cancer Chemother.*, **14**, 119.